# Neighbourhood sociodemographic factors and COVID-19 vaccine uptake in the Netherlands: an ecological analysis

**DOI:** 10.1186/s12889-023-16600-z

**Published:** 2023-09-02

**Authors:** Lisanne J. E. Labuschagne, Naomi Smorenburg, Jan van de Kassteele, Ben Bom, Anne C. de Weerdt, Hester E. de Melker, Susan J. M. Hahné

**Affiliations:** 1 Centre for Infectious Disease Control, National Institute for Public Health and the Environment, Bilthoven, The Netherlands; 2 Public Health and Health Services, National Institute for Public Health and the Environment, Bilthoven, The Netherlands

**Keywords:** Immunisation programmes, COVID-19 vaccines, Ethnicity, Socioeconomic status, Political factors

## Abstract

**Background:**

While overall COVID-19 vaccine uptake is high in the Netherlands, it lags behind in certain subpopulations.

**Aim:**

We aimed to explore the characteristics of groups with lower COVID-19 vaccine uptake at neighbourhood level to inform the strategy to improve uptake and guide research into barriers for vaccination.

**Methods:**

We performed an ecological study using national vaccination register and socio-demographic data at neighbourhood level. Using univariate and multivariable generalized additive models we examined the (potentially non-linear) effect of each determinant on uptake. We focused on those aged 50 years and older, since they are at highest risk of severe disease.

**Results:**

In those over 50 years of age, a higher proportion of individuals with a non-Western migration background and higher voting proportions for right-wing Christian and conservative political parties were at neighbourhood level univariately associated with lower COVID-19 vaccine uptake. In contrast, higher socioeconomic status and higher voting proportions for right-wing liberal, progressive liberal and Christian middle political parties were associated with higher uptake. Multivariable results differed from univariate results in that a higher voting proportion for progressive left-wing political parties was also associated with higher uptake. In addition, with regard to migration background only a Turkish background remained significant.

**Conclusion:**

We identified determinants associated with COVID-19 vaccine uptake at neighbourhood level and observed heterogeneity in uptake between different subpopulations. Since the goal of vaccination is not only to reduce suffering and death by improving the average uptake, but also to reduce health inequity, it is important to focus on subpopulations with lower uptake.

**Supplementary Information:**

The online version contains supplementary material available at 10.1186/s12889-023-16600-z.

## Introduction

The COVID-19 vaccination campaign in the Netherlands started 6 January 2021. The first groups targeted were employees in direct COVID-19 patient care, general practitioners, residents of long term care facilities and other persons living in an institution. In the context of vaccine shortage, the vaccination strategy was to offer vaccination from old to young [1]. By the end of June 2022, the coverage for at least one dose of COVID-19 vaccination for individuals aged 12 years or over was approximately 83%, while 82% was fully vaccinated [2]. Although the overall vaccination coverage was high and the coverage among individuals aged 50 years and older was above 90%, vaccination coverage of younger age groups lagged behind [1, 2].

In addition, the uptake was lower in the four biggest cities, in which the population includes a relatively large number of people with a low socioeconomic status (SES) and/or with a migration background. Previous research indicated that individuals with a migration background and/or low SES have a lower uptake in COVID-19 vaccination [3, 4]. COVID-19 vaccine uptake was also lower in so-called ‘Bible Belt’ municipalities where relatively many orthodox reformed individuals reside, who are known to refuse vaccination more often [1, 5, 6].

In addition to objections to vaccination from religious reasons, previous studies have indicated political preference and trust in the government may also play a role. Conservative right-wing ideology and lack of trust in the government have been previously associated with lower willingness to receive HPV vaccination [6–8] and COVID-19 vaccination [9–12]. The relation between political preference for other political parties and COVID-19 vaccine uptake is unknown, but might also be relevant because of the relation with confidence in government institutions, media and social institutions [13–15] and associations found for HPV-vaccination [6].

To improve our understanding of COVID-19 vaccination behaviour and more specifically to understand which groups are more reluctant to be vaccinated, we performed an ecological study at neighbourhood level. This allowed studying a wide variety of potential determinants which are not yet available for studies at an individual level. The aim of the study was to explore the characteristics of groups that are reluctant to be vaccinated with COVID-19 vaccine at neighbourhood level. This first step will aid the strategy to increase uptake and guide research into barriers for vaccination.

## Methods

### Vaccine uptake

Vaccine uptake was calculated with data from the COVID vaccine Information- and Monitoring System (CIMS). CIMS is a nationwide register including all individuals who are registered in the national population register of the Netherlands. COVID-19 vaccinations are included for vaccinated individuals who have consented for this information to be registered in CIMS. We used CIMS data until April 12^th^ 2022. Approximately 93% of those vaccinated by municipal health services gave consent [16]. Thus, individuals for whom no vaccinations are registered in CIMS are either unvaccinated or did not give consent for their vaccination to be registered. ‘Vaccine uptake’ was defined as having received at least one COVID-19 vaccine. It was not possible to examine coverage (i.e. a completed primary series of COVID-19 vaccination), since we did not have data on SARS-CoV-2 infections, which rendered one dose to be sufficient. Vaccine uptake per neighbourhood was stratified by age group (12–49 and 50 + years). The focus of our analyses was the 50 + age group, since high uptake is particularly important in these older individuals, considering that COVID-19 is more severe at older age.

### Determinants at neighbourhood level

Potential determinants at neighbourhood level were extracted from the publicly available data of Statistics Netherlands (CBS), which included information regarding migration background, socioeconomic status and urbanisation. A neighbourhood is defined as a part of a municipality dominated by a given type of land use or buildings, for instance: industrial area, residential area with high-rise or low-rise buildings (definition CBS) [17]. Results from the National Elections in March 2021 per voting location were available from the Open State Foundation. These results were then translated to voting proportions per neighbourhood (see Supplementary material 1 with a detailed list of political parties) [18]. Distance to nearest vaccination location was calculated as the distance from the centroid of the neighbourhood to the nearest vaccination facility, not including mobile vaccination facilities. Locations of the facilities in use in July 2021, at the peak of the large-scale vaccination campaign, were used. Finally, at the municipality level we obtained information about HPV vaccine uptake in 2020 among girls aged 14 years who were invited for HPV vaccination within the Dutch national immunisation program (NIP) [6]. HPV vaccine uptake was included because uptake for HPV vaccination is also lagging behind in some subpopulations and we were interested to see if the pattern for COVID-19 vaccination was comparable and if HPV vaccine uptake and COVID-19 uptake were correlated.

### Statistical analyses

To examine possible associations between COVID-19 vaccine uptake and each determinant at neighbourhood level, we performed univariate and multivariable generalized additive models with a binomial outcome using a logit-link function. In this way we examined the (potentially non-linear) effect of each determinant on COVID-19 vaccine uptake, while correcting for effects of other determinants. More specifically, we used a quasi-binomial model. The quasi-binomial model is useful when dealing with overdispersion in a binomial setting, as it relaxes the assumption of equidispersion made by the standard binomial model. As such, it takes into account the overdispersion caused by unmeasured neighbourhood specific random variation.

As a first step, we carried out univariate analyses. We subsequently added specific (groups of) variables to the multivariable model to gain more insight into the interrelationships between factors, moving from distal to more proximate factors [19]. The order of the factors in terms or proximity was based on assumptions about causality. It must be noted that our decision about proximity is subjective. First, a model was estimated which only included migration background. In the second model, socioeconomic status was added. Finally, the third model also included urbanisation, distance to nearest vaccination location and voting proportions. Each determinant was included as a penalized spline to model potential non-linear effects. A property of penalised splines is that the effective degrees of freedom (i.e. the number of parameters) is automatically optimised during the fitting procedure, depending on the amount of information in the data. Highly (right) skewed determinants were first transformed to a more uniform or normal scale, e.g. by log- or square root transformation. Effects were presented graphically as odds-ratios of the likelihood to be vaccinated relative to the global average of the specific determinant. In addition, Spearman rank correlations between all determinants, including HPV vaccine uptake, were calculated. All analyses were done using the mgcv package in R [20].

## Results

By April 12^th^ 2022, the national overall COVID-19 vaccine uptake as registered in CIMS was 87.6% among individuals aged 50 years and older and 72.0% among individuals aged 12–49 years. We focused on the results of those aged 50 years and older given the highest risk of severe COVID-19. Analyses of the age group 12–49 years were then compared to the results of those aged 50 years and older.

### Determinants at neighbourhood level associated with COVID-19 vaccine uptake

**Fig. 1:**
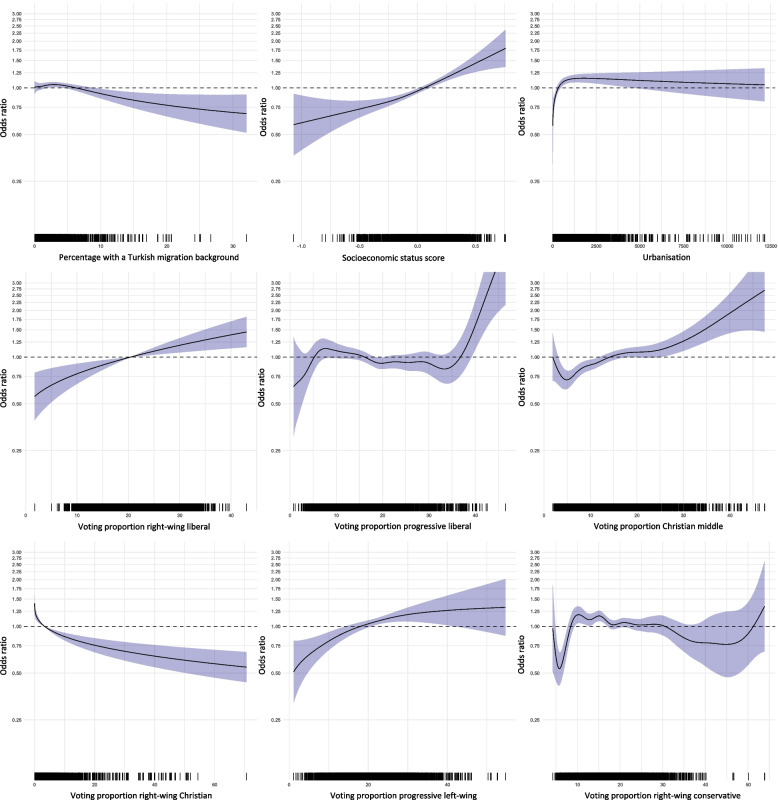
Multivariable binomial logistic regression analyses of the association at neighbourhood level between COVID-19 vaccine uptake and Turkish migration background, socioeconomic status score, urbanisation, and voting proportions for right-wing liberal, progressive liberal, Christian middle, right-wing Christian, progressive left-wing and right-wing conservative political parties (Model 3)

The baseline characteristics of all 3243 populated neighbourhoods in the Netherlands are presented in Table 1. An overview of the direction of the associations and the significance of the determinants in each model for the population aged 50 years and older along with the explained variance (multivariable models) is presented in Table 2. It must be noted, however, that the direction of the association is based on a subjective interpretation of the graphs. We present these graphical results of the final model (model 3) in Fig. 1 whilst those of the univariate analyses, model 1 and 2 are presented in Supplementary Material 2. Based on the graphs in combination with Table 2, the effect sizes and direction of the corresponding association can be determined. Finally, the Spearman rank correlations between all potential determinants on neighbourhood level and COVID-19 vaccine uptake can be found in Supplementary Material 4.

**Table 1:** Baseline characteristics of included neighbourhoods, the Netherlands

	All neighbourhoods^a^ (*n* = 3243) *Median (IQR)*
Non-Western migration background^b^, %	4.0 (2.0 – 10.5)
Moroccan migration background, %	0.2 (0.0 – 1.1)
Antillean migration background, %	0.3 (0.0 – 0.7)
Turkish migration background, %	0.3 (0.0 – 1.2)
Surinamese migration background, %	0.4 (0.0 – 1.0)
Other non-Western migration background, %	2.6 (1.3 – 5.1)
Socioeconomic status score (SES-WOA)^c^	0.14 (-0.01 – 0.25)
Urbanisation^b,d^	581 (167 – 1618)
Distance to nearest vaccination location^e^, m	5258 (2804 – 8470)
Voting proportions^f^	
Right-wing liberal (VVD), %	22.6 (18.2 – 26.9)
Progressive liberal (D66, Volt), %	14.4 (11.1 – 18.2)
Christian middle (CDA, CU), %	13.4 (10.0 – 18.0)
Right-wing Christian (SGP), %	0.4 (0.2 – 1.5)
Progressive left-wing (GL, PvdA, PvdD, SP, DENK), %	20.0 (16.0 – 25.4)
Right-wing conservative (PVV, FvD, JA21), %	18.4 (14.8 – 21.8)
HPV vaccine uptake^g^, %	65.5 (58.0 – 71.6)
COVID-19 vaccine uptake^h^, %	89.0 (85.9 – 91.5)

Data was missing for: Non-western migration background (0.1%); Socioeconomic status score (8.7%); HPV vaccination uptake (3.7%). For all other determinants data was complete

*Abbreviations: CDA* Christian Democratic Appeal, *CU* Christian Union, *D66* Democrats 66, *FvD* Forum for Democracy, *GL* Green Left, *JA21* Right Answer 2021, *PvdA* Labour Party, *PvdD* Party for the Animals, *PVV* Party for Freedom, *SP* Socialist Party, *Volt* Volt Netherlands, *SGP* Reformed Political Party, *VVD* People’s Party for Freedom and Democracy. For explanatory notes on the political parties we refer to Supplementary material 1

^a^A neighbourhood is defined as a part of a municipality dominated by a given type of land use or buildings (i.e., industrial area, residential area with high-rise or low-rise buildings). Neighbourhoods themselves are subdivided into smaller neighbourhood areas. A neighbourhood usually overlaps with a residence or part of a larger residence [17]

^b^Data available from Statistics Netherlands (CBS), 2021

^c^This score represents relative socioeconomic status in comparison with other neighbourhoods based on three elements: Wealth, educational level and labour market participation. A higher score indicates more wealthier/higher educated inhabitants who have worked for a longer period of time. Data available from Statistics Netherlands (CBS), 2019

^d^The average number of addresses within one kilometre radius

^e^Based on information from the Municipal health services on vaccination facilities and calculated as the distance from the core of the neighbourhood to the nearest vaccination facility, not including mobile vaccination facilities. Reference date July 2021

^f^Voting proportions from the National Elections in March 2021 for political parties with at least 2 seats. Data available from Open State Foundation

^g^HPV vaccine uptake in 2020 from the Public Health Services, at municipality level. Includes girls aged 14 years who were invited for and received HPV-vaccination within the Dutch national immunization Program (NIP)

^h^COVID-19 vaccine uptake refers to individuals who had received one dose of COVID-19 vaccine and consented for their data to be shared with the national vaccine register (CIMS). The reference date for vaccine uptake is April 12^th^ 2022

**Table 2:** Results of univariate and multivariable analyses of the associations at neighbourhood level between potential determinants and COVID-19 vaccine uptake among individuals of 50 years and older

	**Univariate**		**Multivariable**					
			**Model 1** (*R*^*2*^ = 17.1%)		**Model 2** (*R*^*2*^ = 20.7%)		**Model 3** (*R*^*2*^ = 41.7%)	
	direction of association	*p*	direction of association	*p*	direction of association	*p*	direction of association	*p*
Non-Western migration background:								
Moroccan	–	< .001	–	< .001	–	< .001	–	0.487
Antillean	–	< .001	–	< .001	0	1.000	0	0.731
Turkish	–	< .001	–	0.001	–	0.006	–	0.026
Surinamese	–	< .001	–	0.012	–	0.017	–	0.171
Other	–	< .001	–	< .001	–	0.020	–	0.137
Higher socioeconomic status	+	< .001			+	< .001	+	< .001
Higher degree of urbanisation	~	< .001					+	< .001
Larger distance to nearest vaccination location	~	< .001					0	0.185
Voting proportions								
Right-wing liberal	+	< .001					+	0.001
Progressive liberal	+	< .001					+	< .001
Christian middle	+	< .001					+	< .001
Right-wing Christian	–	< .001					–	< .001
Progressive left-wing	~	< .001					+	0.002
Right-wing conservative	–	< .001					–	< .001
HPV vaccine uptake	+	< .001						

All associations are at neighbourhood level. For all models the direction of association (positive ( +), negative,(–), no association (0) and mixed ( ~)) and significance of determinants are presented. For multivariable models 1, 2 and 3, the explained variance is also included. The covariate included in model 1 was: migration background. Covariates included in model 2 were: migration background and socioeconomic status. Covariates included in model 3 were: migration background, socioeconomic status. urbanisation, distance to nearest vaccination location and voting proportions

All determinants were significantly associated with COVID-19 vaccine uptake among individuals of 50 years of age and older in univariate analyses (Table 2). With a rising percentage of individuals with any type of non-Western migration background, the odds for vaccine uptake for COVID-19 at the neighbourhood level decreased (Table 2 and Supplementary Fig. 2.1). Higher voting proportions for right-wing Christian and right-wing conservative political parties were also associated with a lower uptake. On the other hand, higher socioeconomic status, higher voting proportions for right-wing liberal, progressive liberal and Christian middle political parties and higher HPV vaccine uptake were univariately associated with higher COVID-19 vaccine uptake. For a higher degree of urbanisation, a higher distance to nearest vaccination location and higher voting proportions for progressive left-wing political parties, the association with COVID-19 vaccine uptake was mixed, meaning that the association was positive or negative depending on the prevalence of the determinant.

In multivariable analyses, the percentages of individuals with all types of non-Western migration background were significantly negatively associated with COVID-19 vaccine uptake in Model 1 (see Table 2 and Supplementary Figure S 2.2). When socioeconomic status was added to the model (Model 2), the association for ‘Antillean’ migration background was no longer significant. In the final model (Model 3), including all potential determinants, for migration background only the association with ‘Turkish’ migration background remained significant.

Higher socioeconomic status was significantly positively associated with COVID-19 vaccine uptake in multivariable analyses (Model 2) and remained significant when other determinants were added in Model 3 (see Fig. 1 Supplementary Figure S 2.2). Higher voting proportions for right-wing liberal, progressive liberal, Christian middle and progressive left-wing political parties were also positively associated with COVID-19 vaccine uptake (Model 3). For higher degree of urbanisation, the association was also significant and positive but only up to a certain level of urbanisation, after which the association stabilised (Model 3, see Fig. 1). Distance to nearest vaccination location was not significantly associated with COVID-19 vaccine uptake (Model 3).

### Differences with age group 12–49 years

**Fig. 2:**
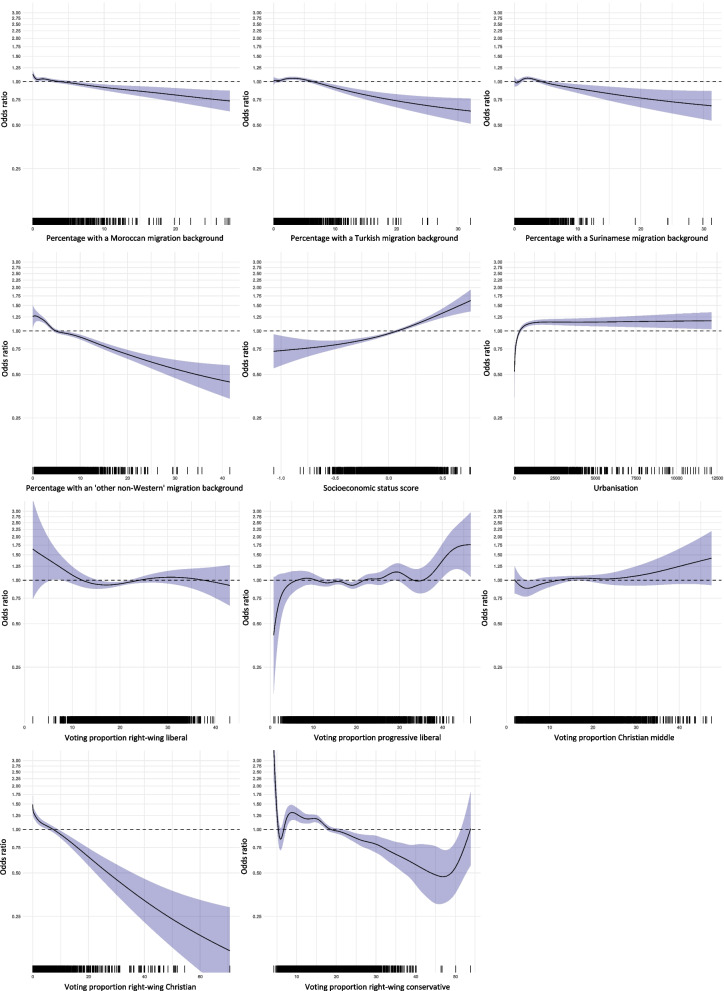
Multivariable binomial logistic regression analyses of the association at neighbourhood level between COVID-19 vaccine uptake and Moroccan, Turkish, Surinamese and ‘other non-Western’ migration background, socioeconomic status score, urbanisation, and voting proportions for right-wing liberal, progressive liberal, Christian middle, right-wing Christian and right-wing conservative political parties (Model 3) (age group 12–49 years)

The results for the age group 12–49 years were mostly similar to those aged 50 years and older (see Table 3, Fig. 2 and Supplementary Material 3), but there are some differences worth mentioning. First of all, while the effects of most non-Western migration backgrounds became non-significant in the final model in those over 50 years of age, in the group aged 12–49 years these associations remained significant, except for Antillean migration background. The directions of the associations were very similar. With respect to voting proportions, the most striking difference was that right-wing liberal voting proportions were negatively associated with vaccine uptake in the final model, while this association was positive for those over 50 years of age. However, in univariate analyses the association was also positive for the younger age group. The association with progressive left-wing voting proportions was not significant. In the final model for those aged 12–49 years, substantially more variance was explained (*R*^2^ = 66.5%) compared to the model for the older age group (*R*^2^ = 41.7%).

**Table 3:** Results of the univariate and multivariable analyses of the associations at neighbourhood level between potential determinants and COVID-19 vaccine uptake among individuals of 12–49 years

	**Univariate**		**Multivariable**					
			**Model 1** (*R*^*2*^ = 36.2%)		**Model 2** (*R*^*2*^ = 42.6%)		**Model 3** (*R*^*2*^ = 66.5%)	
	direction of association	*p*	direction of association	*p*	direction of association	*p*	direction of association	*p*
Non-Western migration background:								
Moroccan	–	< .001	–	< .001	–	< .001	–	< .001
Antillean	–	< .001	–	< .001	–	< .001	0	0.085
Turkish	–	< .001	~	< .001	–	< .001	–	< .001
Surinamese	–	< .001	–	< .001	–	< .001	–	< .001
Other	–	< .001	–	< .001	–	0.003	–	< .001
Higher socioeconomic status	+	< .001			+	< .001	+	< .001
Higher degree of urbanisation	~	< .001					+	< .001
Larger distance to nearest vaccination location	~	< .001					0	0.313
Voting proportions								
Right-wing liberal	+	< .001					–	< .001
Progressive liberal	+	< .001					+	< .001
Christian middle	+	< .001					+	0.018
Right-wing Christian	–	< .001					–	< .001
Progressive left-wing	~	< .001					~	0.181
Right-wing conservative	–	< .001					–	< .001
HPV vaccine uptake	+	< .001						

All associations are at neighbourhood level. For all models, the direction of association (positive ( +), negative,(–), no association (0) and mixed ( ~)) and significance of determinants are presented. For multivariable models 1, 2 and 3, the explained variance is also included. The covariate included in model 1 was: migration background. Covariates included in model 2 were: migration background and socioeconomic status. Covariates included in model 3 were: migration background, socioeconomic status. urbanisation, distance to nearest vaccination location and voting proportions

## Discussion

While various studies reported on determinants for the intention to vaccinate, we were able to study actual vaccine uptake at neighbourhood level and identify possible population subgroups with lower COVID-19 vaccine uptake. Our multivariable results showed that among individuals of 50 years and older vaccine uptake was lower in neighbourhoods with a higher percentage of individuals with a Turkish migration background and higher percentages of voters for right-wing Christian and right-wing conservative political parties. Uptake was clearly higher in neighbourhoods with: a higher socioeconomic status and higher voting proportions for right-wing liberal, progressive liberal, Christian middle and progressive left-wing political parties. Results from the analyses among individuals aged 12–49 years were similar, although overall uptake was lower in this group. Exceptions were, in the multivariable results, stronger significant negative associations for percentage of non-Western migration backgrounds, a non-significant association with voting proportion for progressive left-wing parties and vaccine uptake and a negative instead of a positive association with voting proportion for right-wing liberal parties. However, in univariate analyses the latter association was also positive for the younger age group.

It is clear that vaccine uptake is thus not equally high and subgroups with lower uptake exist. Our results are largely in line with small survey studies investigating vaccination willingness or hesitancy that have been performed in the Netherlands, indicating that individuals with a non-Western migration background and/or a lower socioeconomic status are less likely to be vaccinated against COVID-19 [3, 4]. Our findings are also consistent with earlier studies in the Netherlands concerning other vaccines. Parents’ country of birth, percentage of votes for the conservative Christian reformed party and low educational level have been associated with both lower HPV [6] and MenACWY-vaccine uptake [21]. In addition, in our study, HPV-vaccination background was strongly positively associated with COVID-19 vaccine uptake. Determinants for COVID-19 vaccination and HPV-vaccination are therefore likely similar.

International studies on willingness to vaccinate report findings consistent with ours. In a large systematic review about factors that influence unwillingness or hesitancy to vaccinate against COVID-19 among older individuals, the likelihood of being unvaccinated was significantly higher in ethnic minority groups, or individuals with a low education or low income [22]. Multiple studies have confirmed that older individuals [23–25] and individuals with higher socioeconomic status [23, 24, 26–28] were more likely to report the intention to be vaccinated against COVID-19. Being unemployed [29], having an ethnic minority status [30] and living in disadvantaged areas [31] were factors associated with lower willingness to be vaccinated against COVID-19.

Non-Western migration background, and more specifically Turkish migration background was on neighbourhood level an important factor associated with lower COVID-19 vaccine uptake. Turkish background remained significant in the final model, while for the other migration backgrounds the model indicated that the effect was mediated by the other determinants included (i.e. voting and degree of urbanisation). Generally, trust in the government, vaccine safety and perceived risk of infection and disease severity are factors that are related to lower vaccine uptake in the general population, and in migrant groups in particular [4]. Other additional possible barriers that migrants might experience are language barriers, cultural or religious barriers, practical barriers and fear of stigmatisation [4, 32]. Cultural background and socioeconomic status might be underlying factors. Similar barriers might be experienced by other subgroups with lower vaccine uptake, including those with lower trust in the government (right-wing conservative parties) and strong religious motivations (right-wing Christian). Future studies should explore these reasons more in depth and at an individual level.

Results on urbanisation were more difficult to interpret. As the degree of urbanisation increased, the likelihood to be vaccinated against COVID-19 first increased. However, at a higher level this effect stabilised. Neighbourhoods with a higher degree of urbanisation thus had higher COVID-19 vaccine uptake compared to very unpopulated areas, but after a certain threshold, vaccine uptake did not differ. Previous studies have found an association between lower vaccine uptake and living in urban areas [33]. Distance to nearest vaccination location was not significant. It should be noted, however, that we were not able to include mobile vaccination locations. In addition, distance was highly correlated with urbanisation and non-Western migration background, which might have rendered it redundant in the multivariable analyses.

The main strength of our study is that we were able to investigate COVID-19 vaccine uptake directly in contrast to previous studies that only concerned individuals’ willingness to be vaccinated before the vaccine was actually available. In addition, this study demonstrates that information on neighbourhood level can be useful for countries that do not have a national vaccination registry. Our study also has some limitations. We performed ecological analyses at neighbourhood level, which requires the results to be interpreted with caution due to the potential for ecologic fallacy. In addition, since we only had data on individuals who had consented for their vaccination status to be shared with the national vaccine register (CIMS), we were essentially investigating determinants for vaccine uptake *and* informed consent. COVID-19 vaccine uptake in this study is therefore an underestimation of the true uptake and assessment of determinants may be affected by bias. However, we do not have much information about what characteristics are related to informed consent and are therefore unable to estimate the effect and direction of this bias. We do know that older individuals are more likely to provide informed consent than younger individuals (personal communication, S. McDonald), and vaccine uptake is also higher in older individuals. However, since we do not have information on other characteristics of the non-consent group we cannot investigate the extent of the bias introduced. Regarding distance to nearest vaccination location, we were not able to include mobile vaccination facilities. However, mobile vaccination units were only used sporadically at the very end of the vaccination campaign, starting November 2021. Even though they were primarily used in neighbourhoods with lower uptake, we do not expect that not including these facilities substantially influenced our results, because people already had had ample time to be vaccinated, especially the older age group. A final limitation is that the age-demography of neighbourhoods was not available, which might have influenced our results. Since vaccine uptake is lower in younger age groups, neighbourhoods that included many younger individuals would have a lower uptake, but unfortunately we were unable to study this.

### Implications for policy and further research

This study has provided some first insights into what groups at neighbourhood level lag behind in the COVID-19 vaccine uptake and should be at the focus of future vaccination campaigns. Notably, neighbourhoods with higher proportions of individuals with a non-Western migration background and higher voting proportions for right-wing Christian and conservative political parties require more attention. It might be useful for future campaigns to target these subgroups and use extra resources, for example through key community figures. Future research should aim to assess if the determinants for lower uptake at neighbourhood level correspond with those at an individual level. In addition, further analyses should be aimed towards the specific drivers and barriers of vaccine uptake among these subgroups with low uptake.

## Conclusion

Even though in the Netherlands overall COVID-19 vaccine uptake is high, we observed important heterogeneity between different subpopulations at neighbourhood level. Our results require further investigation and this study can therefore be considered as a first step to guide further research into what determinants might play a role in COVID-19 vaccine uptake and what population subgroups require more attention in vaccination campaigns. Further research on the role of the current determinants in COVID-19 vaccine uptake at an individual level and underlying reasons for not being vaccinated is recommended and underway. This is of key importance, since the goal of the vaccination programme is to not only prevent suffering and death by improving the average uptake, but also to reduce health inequity [34].

### Supplementary Information

**Additional file 1: Table S1.1.** Political parties included in the in the Dutch House of Representatives in 2021. **Figure S2.1.** Univariate binomial logistic regression analyses of the association at neighbourhood level between COVID-19 vaccine uptake and Moroccan, Antillean, Turkish, Surinamese, and ‘ other non-Western’ migration background, socioeconomic status score, urbanisation, distance to nearest vaccination location, voting proportions for right-wing liberal, progressive liberal, Christian middle, right-wing Christian, progressive left-wing and right-wing conservative political parties and HPV vaccine uptake. **Figure S2.2.** Multivariable binomial logistic regression analyses of the association at neighbourhood level between COVID-19 vaccine uptake and Moroccan, Antillean, Turkish, Surinamese, and ‘ other non-Western’ migration background, socioeconomic status score and distance to nearest vaccination location (models 1, 2 and 3). **Figure S3.1.** Univariate binomial logistic regression analyses of the association at neighbourhood level between COVID-19 vaccine uptake and Moroccan, Turkish, Surinamese, and ‘ other non-Western’ migration background, socioeconomic status score, urbanisation, distance to nearest vaccination location, voting proportions for right-wing liberal, progressive liberal, Christian middle, right-wing Christian, progressive left-wing and right-wing conservative political parties and HPV vaccine uptake (age group 12-49 years). **Figure S3.2.** Multivariable binomial logistic regression analyses of the association at neighbourhood level between COVID-19 vaccine uptake and Moroccan, Antillean, Turkish, Surinamese, and ‘ other non-Western’ migration background, socioeconomic status score, distance to nearest vaccination location and voting proportion for progressive left-wing political parties (models 1, 2 and 3) (age group 12-49 years). **Figure S4.1.** Correlations between all potential determinants, HPV vaccine uptake and COVID-19 vaccine uptake at neighbourhood level (age group 50+ years). **Figure S4.2.** Correlations between all potential determinants, HPV vaccine uptake and COVID-19 vaccine uptake at neighbourhood level (age group 12-49 years).

## Data Availability

Data about determinants at neighbourhood level are publicly available at Statistics Netherlands (CBS) [17] and the Open State Foundation [18]. Data about vaccine uptake are only available at municipal level from RIVM open data (https://data.rivm.nl/covid-19/), but can become available at neighbourhood level from the corresponding author on reasonable request.

## References

[1] Pluijmakers, AJM, de Melker, HE. The National Immunisation Programme in the Netherlands. Surveillance and developments in 2020–2021. RIVM, 2021. 10.21945/RIVM-2021-0055.

[2] Rijksinstituut voor Volksgezondheid en Milieu (RIVM). Archief wekelijkse update vaccinatiecijfers 2021 [Archive weekly update number of vaccinations]. Bilthoven: RIVM. Available from: https://www.rivm.nl/covid-19-vaccinatie/archief-wekelijkse-update-vaccinatiecijfers-2021. [Accessed: 30 June 2022].

[3] Rijksinstituut voor Volksgezondheid en Milieu (RIVM) Corona Gedragsunit. Deelname aan COVID-19 vaccinatie; Stand van zaken, factoren die van invloed zijn, verwachtingen en beleidsimplicaties – kennisupdate. Bilthoven: RIVM. 2021. Available from: https://www.rivm.nl/documenten/deelname-aan-covid-19-vaccinatie-stand-van-zaken.

[4] Rijksinstituut voor Volksgezondheid en Milieu (RIVM) Corona Gedragsunit. Vaccinatiebereidheid COVID-19 onder groepen met een migratieachtergrond; verkenning van beïnvloedende factoren en strategieën voor communicatie en beleid. Bilthoven: RIVM; 2021. Available from: https://www.rivm.nl/documenten/vaccinatiebereidheid-covid-19-onder-groepen-met-migratieachtergrond.

[5] Ruijs WL, Hautvast JL, van der Velden K, de Vos S, Knippenberg H, Hulscher ME (2011). Religious subgroups influencing vaccination coverage in the Dutch Bible belt: an ecological study. BMC Public Health.

[6] de Munter AC, Klooster TM, van Lier A, Akkermans R, de Melker HE, Ruijs WLM (2021). Determinants of HPV-vaccination uptake and subgroups with a lower uptake in the Netherlands. BMC Public Health.

[7] Marlow LA, Waller J, Wardle J (2007). Trust and experience as predictors of HPV vaccine acceptance. Hum Vaccin.

[8] Gefenaite G, Smit M, Nijman HW, Tami A, Drijfhout IH, Pascal A, Postma MJ, Wolters BA, van Delden JJ, Wilschut JC, Hak E (2012). Comparatively low attendance during Human Papillomavirus catch-up vaccination among teenage girls in the Netherlands: Insights from a behavioral survey among parents. BMC Public Health.

[9] Wang Q, Yang L, Jin H, Lin L (2021). Vaccination against COVID-19: A systematic review and meta-analysis of acceptability and its predictors. Prev Med.

[10] Wollebæk D, Fladmoe A, Steen-Johnsen K, Ihlen Ø (2022). Right-wing ideological constraint and vaccine refusal: The case of the COVID-19 vaccine in Norway. Scan Polit Stud.

[11] Viswanath K, Bekalu M, Dhawan D, Pinnamaneni R, Lang J, McLoud R (2021). Individual and social determinants of COVID-19 vaccine uptake. BMC Public Health.

[12] Troiano G, Nardi A (2021). Vaccine hesitancy in the era of COVID-19. Public Health.

[13] Centraal Bureau voor de Statistiek (CBS). Het profiel van het electoraat in 2012 Den Haag. 2017. Available from: https://www.cbs.nl/nl-nl/achtergrond/2017/08/het-profiel-van-het-electoraat-in-2012.

[14] Sociaal en Cultureel Planbureau (SCP). Eigentijdse ongelijkheid. De postindustriële klassenstructuur op basis van vier typen kapitaal. Verschil in Nederland 2023. Den Haag: SCP; 2023. Available from: https://www.scp.nl/publicaties/publicaties/2023/03/07/eigentijdse-ongelijkheid.

[15] Wendelmoet Boersema, Romana Abels. Onderzoek Kieskompas: Complotten? Kiezers van FvD, PVV en 50Plus zweren erbij 2019 [07–12–2019]. Available from: https://www.trouw.nl/binnenland/complotten-kiezersvan-fvd-pvv-en-50plus-zweren-erbij~b2138a0d/?utm_campaign=shared_earned&utm_medium=social&utm_source=email.

[16] Rijksinstituut voor Volksgezondheid en Milieu (RIVM), Centrum Epidemiologie (EPI) in samenwerking met het Centraal Bureau voor de Statistiek (CBS). COVID-19 vaccinatieopkomst van werknemers naar bedrijfsklasse, met bijzondere aandacht voor de zorgsector. Bilthoven: RIVM. 2022. Available from: https://www.rivm.nl/publicaties/covid-19-vaccinatieopkomst-van-werknemers-naar-bedrijfsklasse-met-bijzondere-aandacht.

[17] Centraal Bureau voor de Statistiek (CBS). Toelichting kerncijfers wijken en buurten. Den Haag: CBS. Available from: https://www.cbs.nl/nl-nl/maatwerk/2021/31/kerncijfers-wijken-en-buurten-2021. [Accessed: 12 April 2022].

[18] PDC Informatie Architectuur. Partijen in Eerste en Tweede Kamer. Available from: https://www.parlement.com/id/vh8lnhrpfxut/partijen_in_tweede_en_eerste_kamer. [Accessed: 30 June 2022].

[19] Victora CG, Huttly SR, Fuchs SC, Olinto MT (1997). The role of conceptual frameworks in epidemiological analysis: a hierarchical approach. Int J Epidemiol.

[20] Wood SN (2004). Stable and efficient multiple smoothing parameter estimation for generalized additive models. J. Amer. Statist. Ass.

[21] Lima PDOB, van Lier A, de Melker H, Ferreira JA, van Vliet H, Knol MJ (2020). MenACWY vaccination campaign for adolescents in the Netherlands: Uptake and its determinants. Vaccine.

[22] Veronese N, Saccaro C, Demurtas J, Smith L, Dominguez LJ, Maggi S, Barbagallo M (2021). Prevalence of unwillingness and uncertainty to vaccinate against COVID-19 in older people: A systematic review and meta-analysis. Ageing research reviews.

[23] La Vecchia C, Negri E, Alicandro G, Scarpino V (2020). Attitudes towards influenza vaccine and a potential COVID-19 vaccine in Italy and differences across occupational groups, September 2020. La Medicina del lavoro..

[24] Soares P, Rocha JV, Moniz M, Gama A, Laires PA, Pedro AR (2021). Factors associated with COVID-19 vaccine hesitancy. Vaccines.

[25] Kourlaba G, Kourkouni E, Maistreli S, Tsopela CG, Molocha NM, Triantafyllou C (2021). Willingness of Greek general population to get a COVID-19 vaccine. Global health research and policy.

[26] Afifi TO, Salmon S, Taillieu T, Stewart-Tufescu A, Fortier J, Driedger SM (2021). Older adolescents and young adults willingness to receive the COVID-19 vaccine: Implications for informing public health strategies. Vaccine.

[27] Schwarzinger M, Watson V, Arwidson P, Alla F, Luchini S (2021). COVID-19 vaccine hesitancy in a representative working-age population in France: a survey experiment based on vaccine characteristics. The Lancet Public Health.

[28] Dodd RH, Cvejic E, Bonner C, Pickles K, McCaffery KJ, Ayre J (2021). Willingness to vaccinate against COVID-19 in Australia. Lancet Infect Dis.

[29] Malik AA, McFadden SM, Elharake J, Omer SB (2020). Determinants of COVID-19 vaccine acceptance in the US. EClinicalMedicine.

[30] Kreps S, Prasad S, Brownstein JS, Hswen Y, Garibaldi BT, Zhang B, Kriner DL (2020). Factors associated with US adults’ likelihood of accepting COVID-19 vaccination. JAMA Netw Open.

[31] Edwards B, Biddle N, Gray M, Sollis K (2021). COVID-19 vaccine hesitancy and resistance: Correlates in a nationally representative longitudinal survey of the Australian population. PloS one.

[32] Crawshaw AF, Farah Y, Deal A, Rustage K, Hayward SE, Carter J (2022). Defining the determinants of vaccine uptake and undervaccination in migrant populations in Europe to improve routine and COVID-19 vaccine uptake: a systematic review. Lancet Infect Dis.

[33] Wang G, Yao Y, Wang Y (2023). Determinants of COVID-19 vaccination status and hesitancy among older adults in China. Nat Med.

[34] Hahné S, Bollaerts K, Farrington P (2021). Vaccination Programmes: Epidemiology, Monitoring.

