# Supplementary material for: Neighbourhood sociodemographic factors and COVID-19 vaccine uptake in the Netherlands: an ecological analysis
Source: BMC Public Health. 2023 Sep 2;23:1696. doi: 10.1186/s12889-023-16600-z (PMC10474671; doi:10.1186/s12889-023-16600-z)

Supplementary materials

This supplementary material is hosted by *Eurosurveillance* as supporting information alongside the article Determinants of COVID-19 vaccination uptake in the Netherlands: an ecological analysis, on behalf of the authors, who remain responsible for the accuracy and appropriateness of the content. The same standards for ethics, copyright, attributions and permissions as for the article apply. Supplements are not edited by *Eurosurveillance* and the journal is not responsible for the maintenance of any links or email addresses provided therein.

Table S1.1. Political parties included in the in the Dutch House of Representatives in 2021

Abbreviation in Dutch	Party's name in English	Political movement / political spectrum
VVD	People's Party for Freedom and Democracy	Right-wing liberal party with more progressive positions in ethical matters
D66	Democrats 66	Reformist social-liberal party
Volt	Volt Netherlands	Social-liberal pro-Europeanism party
CDA	Christian Democratic Appeal	Christian-inspired party at the center of the political spectrum
CU	Christian Union	Christian party, with progressive positions in the social and ecological field and conservative positions on ethical issues
SGP	Reformed Political Party	Conservative Christian (Reformed) party that wants to conduct politics strictly according to Biblical standards
GL	Green Left	Progressive party which attaches great importance to sustainability
PvdA	Labor Party	Progressive, social-democratic party
PvdD	The Party for the Animals	Testimonial party with as main goals animal rights and animal welfare
SP	Socialist Party	Socialist, Eurosceptic party which has a strong local, action-oriented basis
DENK	Denk	Movement for migrants and a "tolerant and solidary society"
PVV	Party for Freedom	Populist party with both conservative, liberal "right" and "left" views
FvD	Forum for Democracy	Conservative, right-wing populist Eurosceptic political party
JA21	Right Answer 2021	Conservative right-wing liberal party, split from FvD

Note: Political movements were grouped in the following manner: Right-wing liberal: VVD; Progressive liberal: D66, Volt; Christian middle: CDA, CU; Right-wing Christian: SGP; Progressive left-wing: GL, PvdA, PvdD, SP, DENK; Right-wing conservative: PVV, FvD, JA21.

Figure S2.1. **Univariate binomial logistic regression analyses of the association at neighbourhood level between COVID-19 vaccine uptake and Moroccan, Antillean, Turkish, Surinamese, and ‘ other non-Western’ migration background, socioeconomic status score, urbanisation, distance to nearest vaccination location, voting proportions for right-wing liberal, progressive liberal, Christian middle, right-wing Christian, progressive left-wing and right-wing conservative political parties and HPV vaccine uptake.**

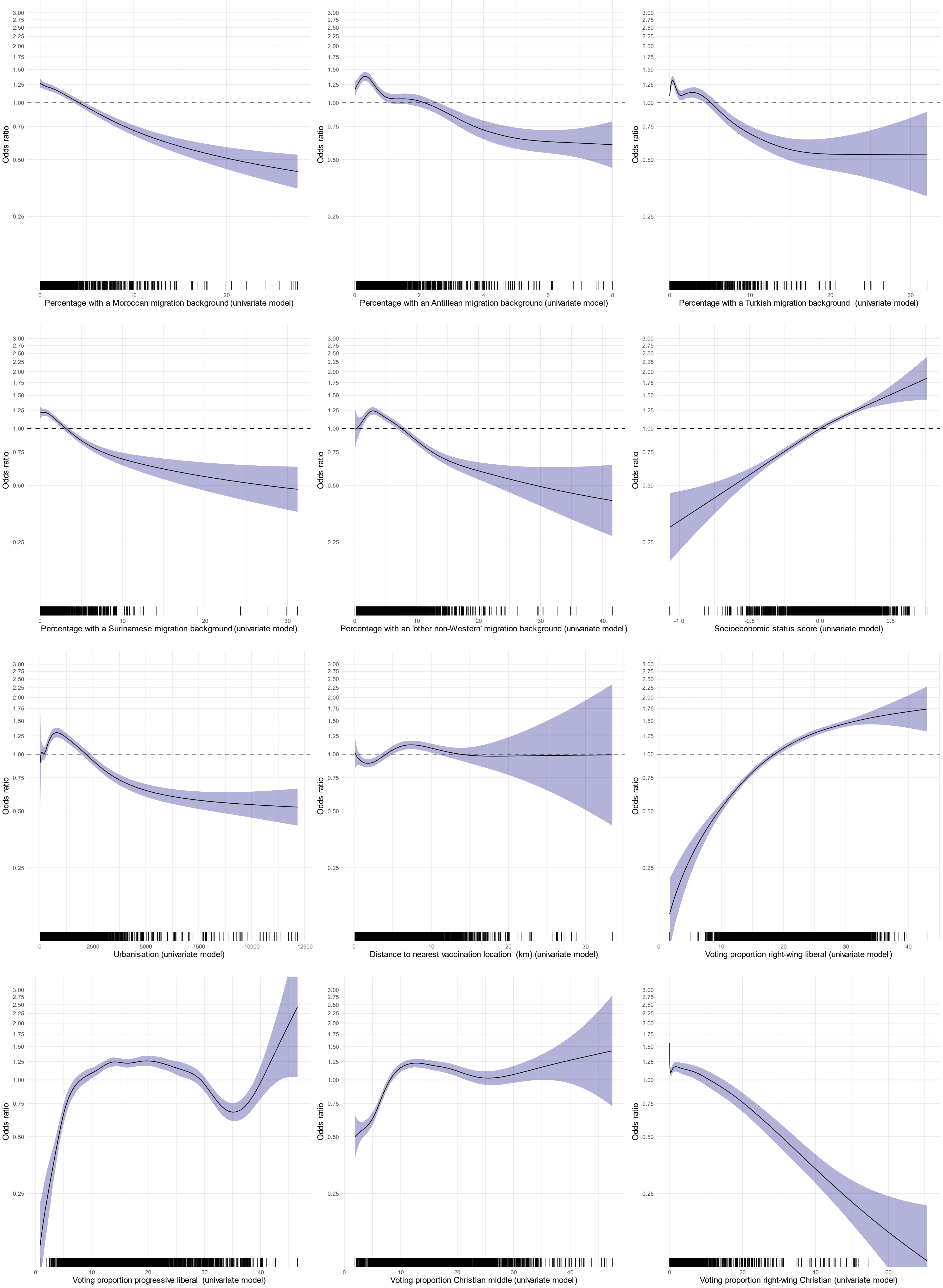
Odds ratio
Percentage with a Moroccan migration background (univariate model)
Percentage with an Antillean migration background (univariate model)
Percentage with a Turkish migration background (univariate model)
Percentage with a Surinamese migration background (univariate model)
Percentage with an 'other non-Western' migration background (univariate model)
Socioeconomic status score (univariate model)
Urbanisation (univariate model)
Distance to nearest vaccination location (km) (univariate model)
Voting proportion right-wing liberal (univariate model)
Voting proportion progressive liberal (univariate model)
Voting proportion Christian middle (univariate model)
Voting proportion right-wing Christian (univariate model)

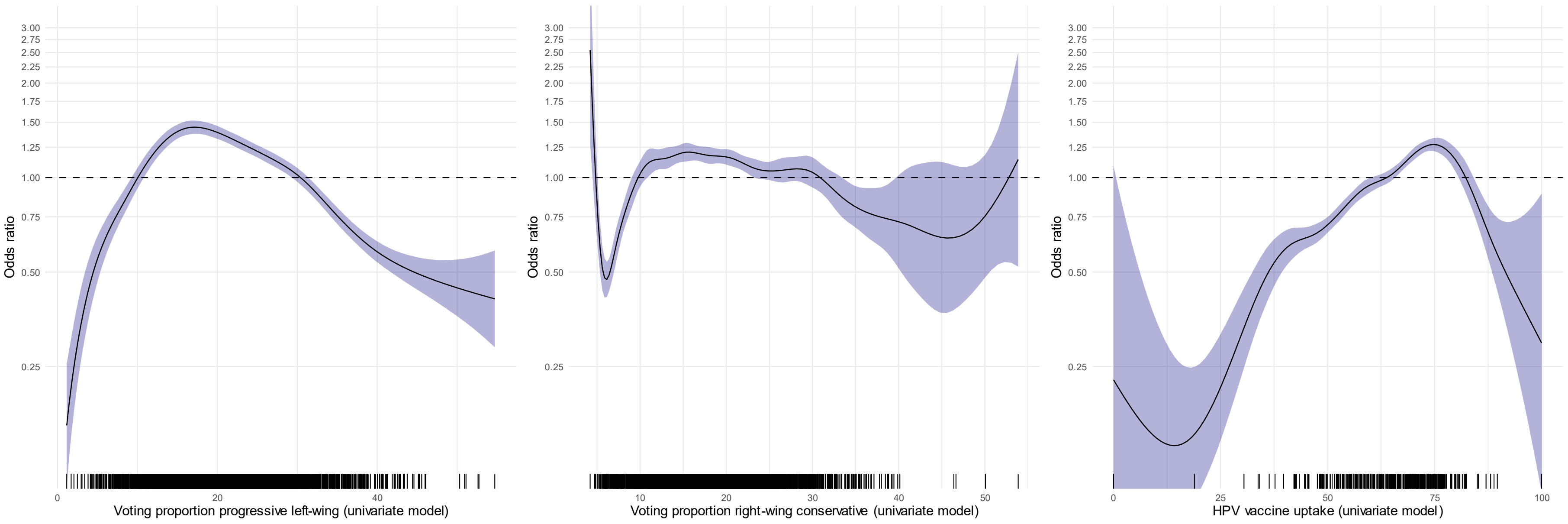
Odds ratio
3.00
2.75
2.50
2.25
2.00
1.75
1.50
1.25
1.00
0.75
0.50
0.25
0
20
40
Voting proportion progressive left-wing (univariate model)
10
20
30
40
50
Voting proportion right-wing conservative (univariate model)
0
25
50
75
100
HPV vaccine uptake (univariate model)

Figure S2.2. **Multivariable binomial logistic regression analyses of the association at neighbourhood level between COVID-19 vaccine uptake and Moroccan, Antillean, Turkish, Surinamese, and ‘ other non-Western’ migration background, socioeconomic status score and distance to nearest vaccination location (models 1, 2 and 3).**

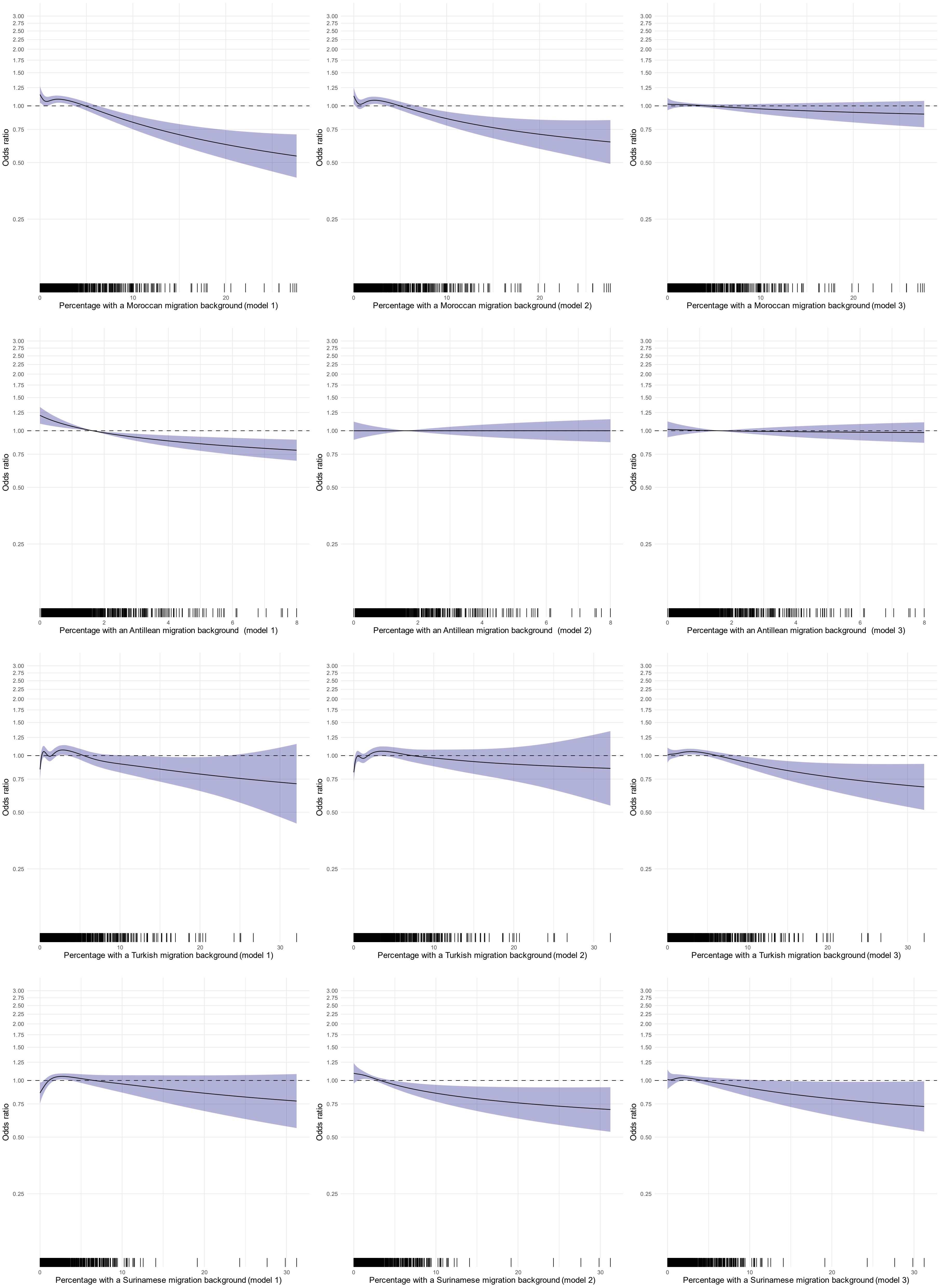
Odds ratio
3.00
2.75
2.50
2.25
2.00
1.75
1.50
1.25
1.00
0.75
0.50
0.25
Percentage with a Moroccan migration background (model 1)
Percentage with a Moroccan migration background (model 2)
Percentage with a Moroccan migration background (model 3)
Percentage with an Antillean migration background (model 1)
Percentage with an Antillean migration background (model 2)
Percentage with an Antillean migration background (model 3)
Percentage with a Turkish migration background (model 1)
Percentage with a Turkish migration background (model 2)
Percentage with a Turkish migration background (model 3)
Percentage with a Surinamese migration background (model 1)
Percentage with a Surinamese migration background (model 2)
Percentage with a Surinamese migration background (model 3)

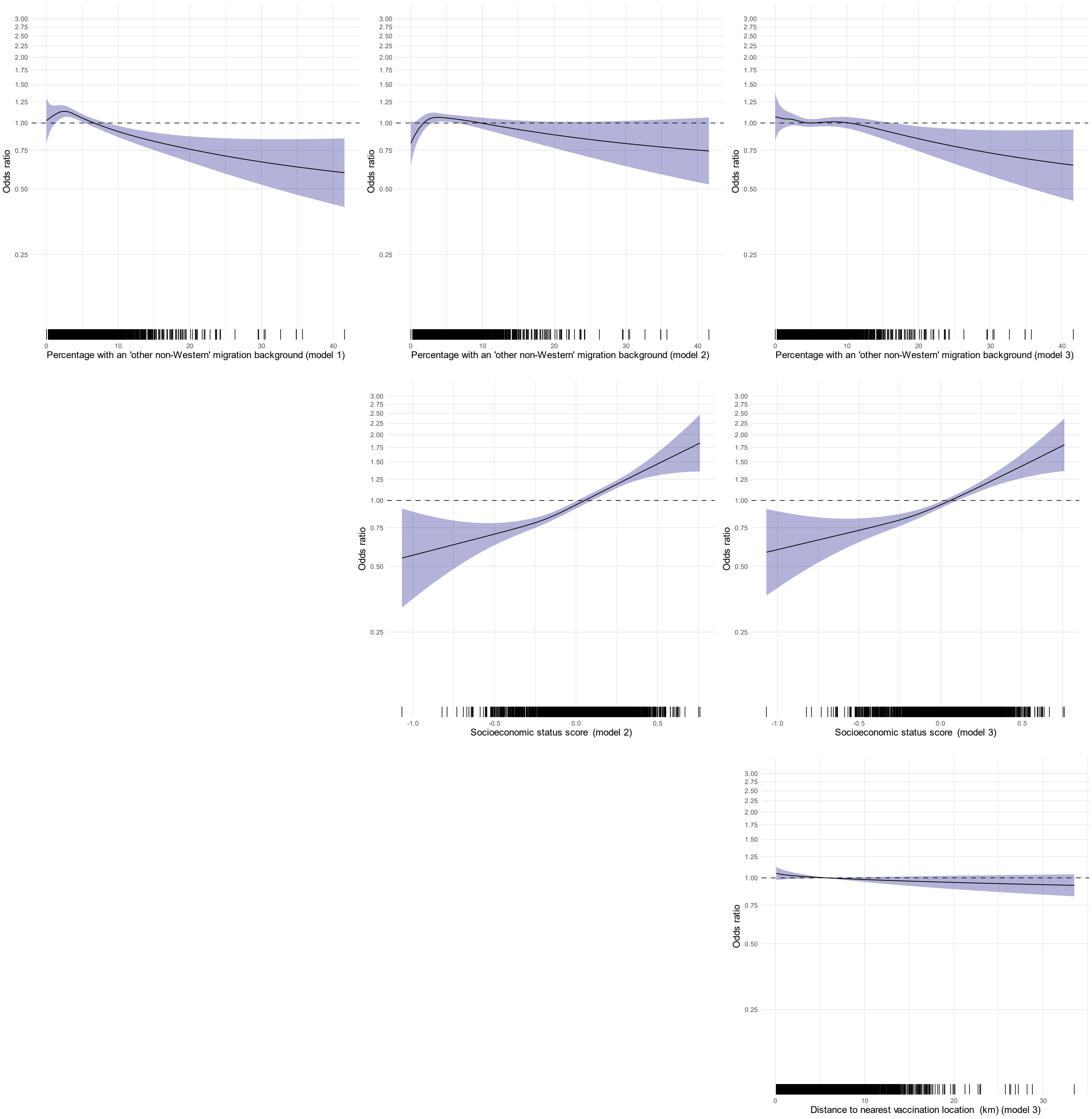
Odds ratio
3.00
2.75
2.50
2.25
2.00
1.75
1.50
1.25
1.00
0.75
0.50
0.25
Percentage with an 'other non-Western' migration background (model 1)
Percentage with an 'other non-Western' migration background (model 2)
Percentage with an 'other non-Western' migration background (model 3)
Socioeconomic status score (model 2)
Socioeconomic status score (model 3)
Distance to nearest vaccination location (km) (model 3)

Figure S3.1. **Univariate binomial logistic regression analyses of the association at neighbourhood level between COVID-19 vaccine uptake and Moroccan, Turkish, Surinamese, and ‘ other non-Western’ migration background, socioeconomic status score, urbanisation, distance to nearest vaccination location, voting proportions for right-wing liberal, progressive liberal, Christian middle, right-wing Christian, progressive left-wing and right-wing conservative political parties and HPV vaccine uptake (age group 12-49 years).**

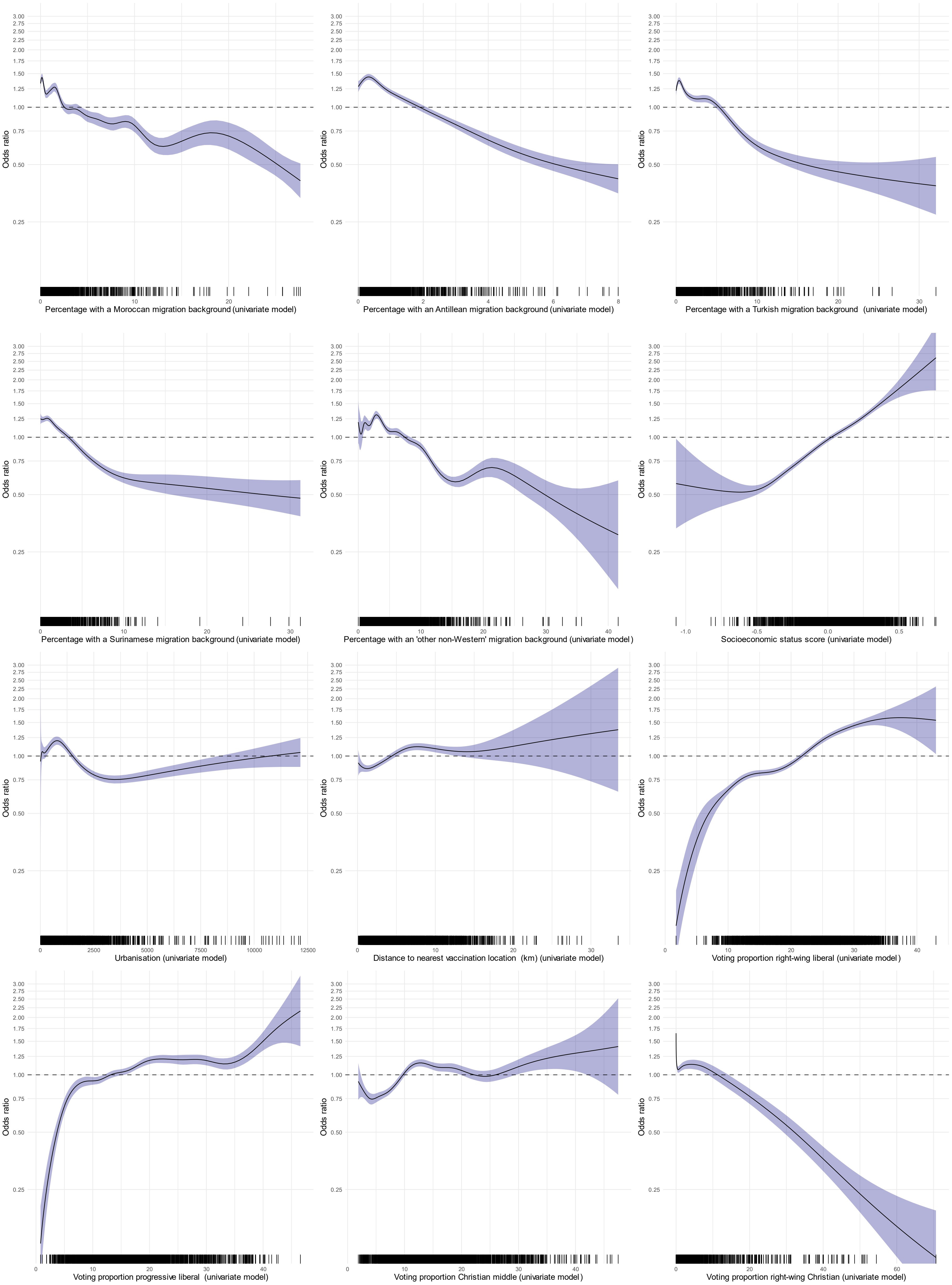
Odds ratio
3.00
2.75
2.50
2.25
2.00
1.75
1.50
1.25
1.00
0.75
0.50
0.25
Percentage with a Moroccan migration background (univariate model)
Percentage with an Antillean migration background (univariate model)
Percentage with a Turkish migration background (univariate model)
Percentage with a Surinamese migration background (univariate model)
Percentage with an 'other non-Western' migration background (univariate model)
Socioeconomic status score (univariate model)
Urbanisation (univariate model)
Distance to nearest vaccination location (km) (univariate model)
Voting proportion right-wing liberal (univariate model)
Voting proportion progressive liberal (univariate model)
Voting proportion Christian middle (univariate model)
Voting proportion right-wing Christian (univariate model)

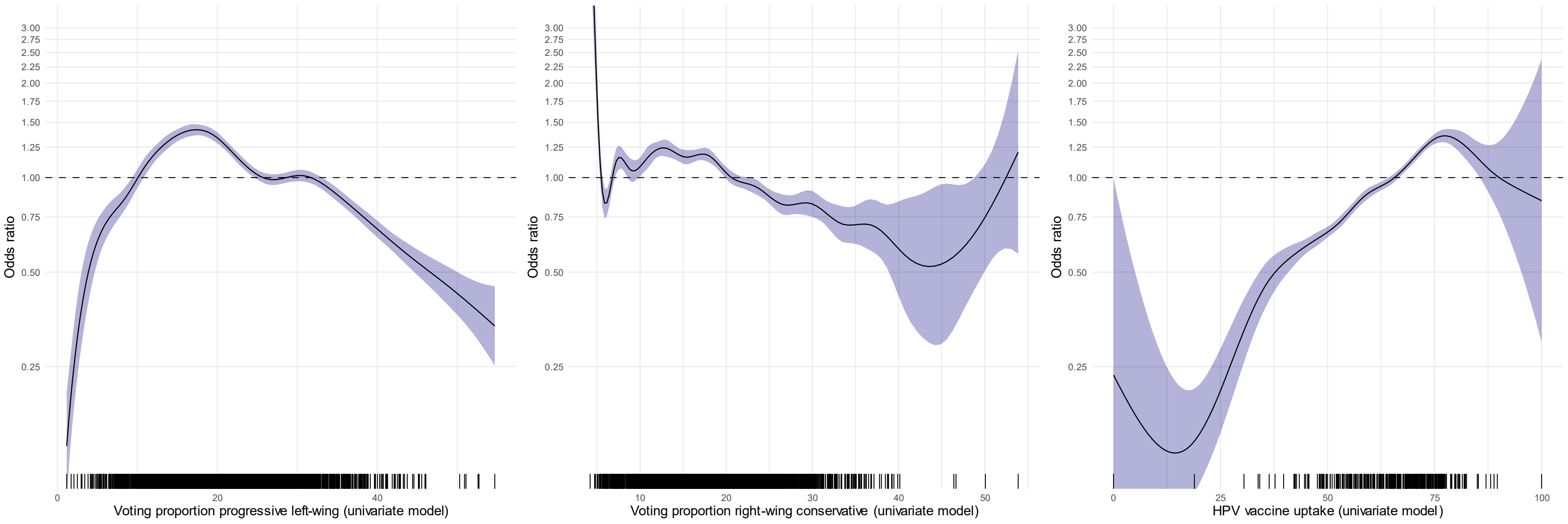
Odds ratio
Voting proportion progressive left-wing (univariate model)
Voting proportion right-wing conservative (univariate model)
HPV vaccine uptake (univariate model)

Figure S3.2. **Multivariable binomial logistic regression analyses of the association at neighbourhood level between COVID-19 vaccine uptake and Moroccan, Antillean, Turkish, Surinamese, and ‘ other non-Western’ migration background, socioeconomic status score, distance to nearest vaccination location and voting proportion for progressive left-wing political parties (models 1, 2 and 3) (age group 12-49 years).**

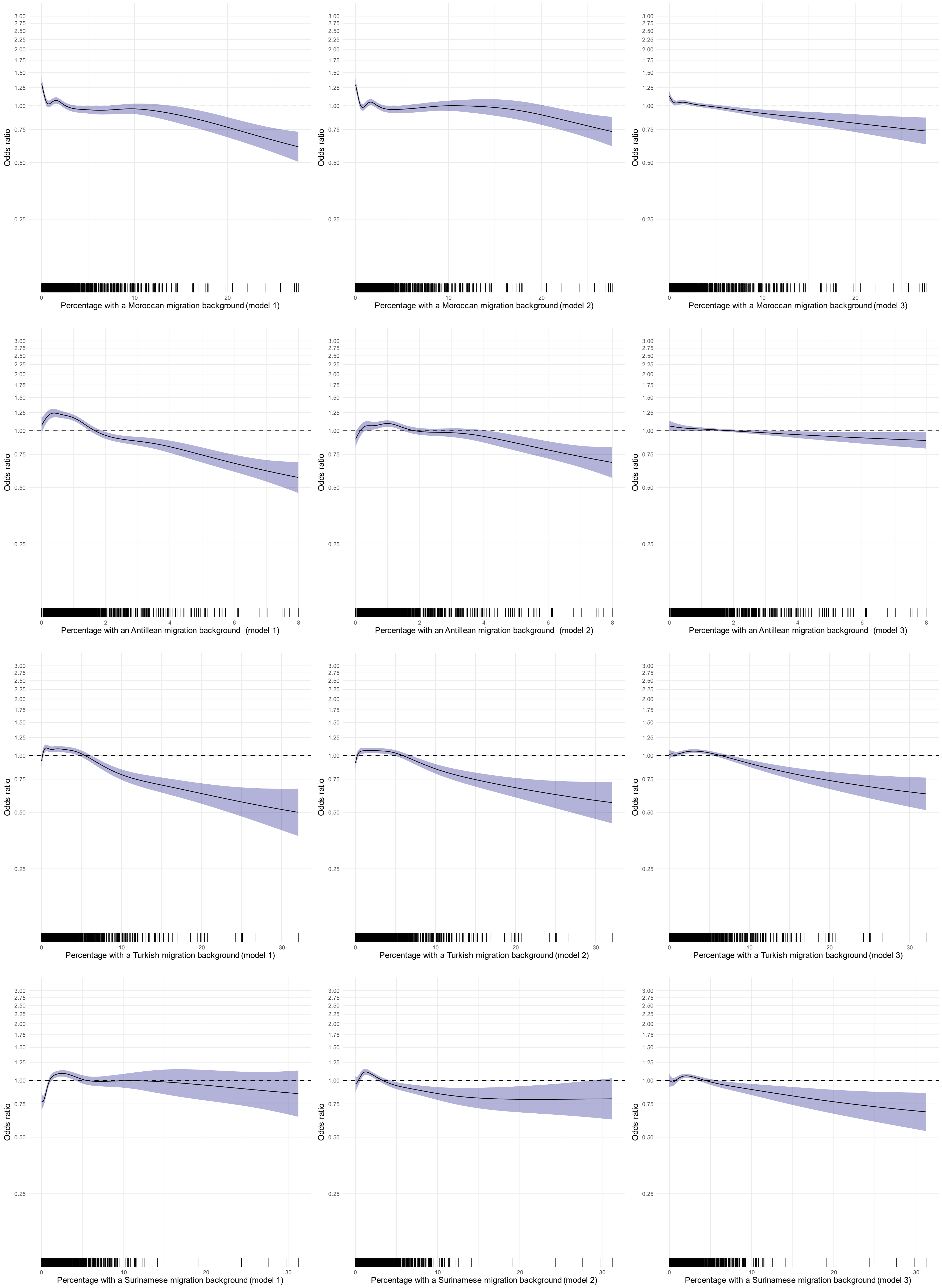
Odds ratio
3.00
2.75
2.50
2.25
2.00
1.75
1.50
1.25
1.00
0.75
0.50
0.25
Percentage with a Moroccan migration background (model 1)
Percentage with a Moroccan migration background (model 2)
Percentage with a Moroccan migration background (model 3)
Percentage with an Antillean migration background (model 1)
Percentage with an Antillean migration background (model 2)
Percentage with an Antillean migration background (model 3)
Percentage with a Turkish migration background (model 1)
Percentage with a Turkish migration background (model 2)
Percentage with a Turkish migration background (model 3)
Percentage with a Surinamese migration background (model 1)
Percentage with a Surinamese migration background (model 2)
Percentage with a Surinamese migration background (model 3)

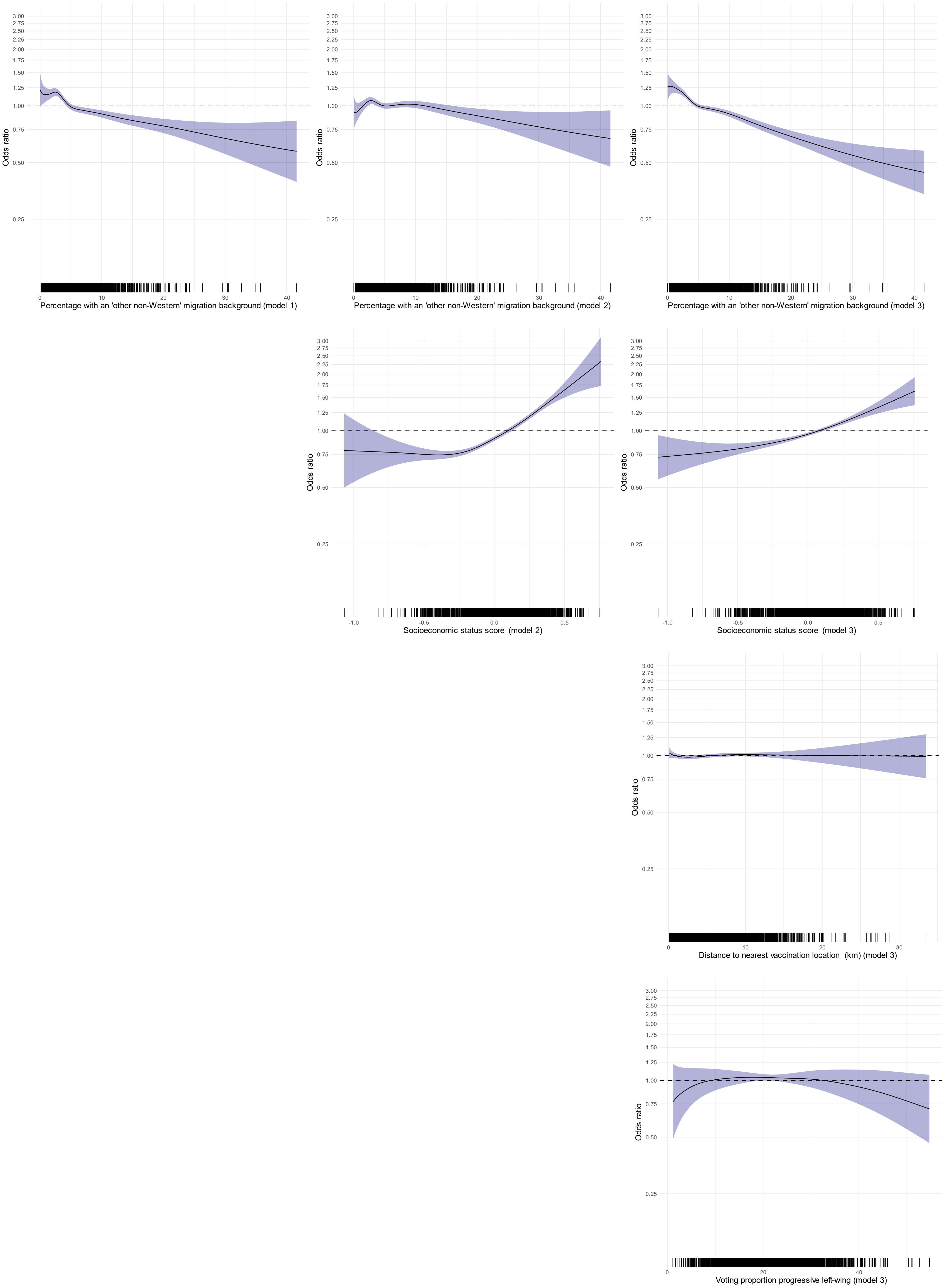
Odds ratio
Percentage with an 'other non-Western' migration background (model 1)
Odds ratio
Percentage with an 'other non-Western' migration background (model 2)
Odds ratio
Percentage with an 'other non-Western' migration background (model 3)
Odds ratio
Socioeconomic status score (model 2)
Odds ratio
Socioeconomic status score (model 3)
Odds ratio
Distance to nearest vaccination location (km) (model 3)
Odds ratio
Voting proportion progressive left-wing (model 3)

Figure S4.1. **Correlations between all potential determinants, HPV vaccine uptake and COVID-19 vaccine uptake at neighbourhood level (age group 50+ years).**

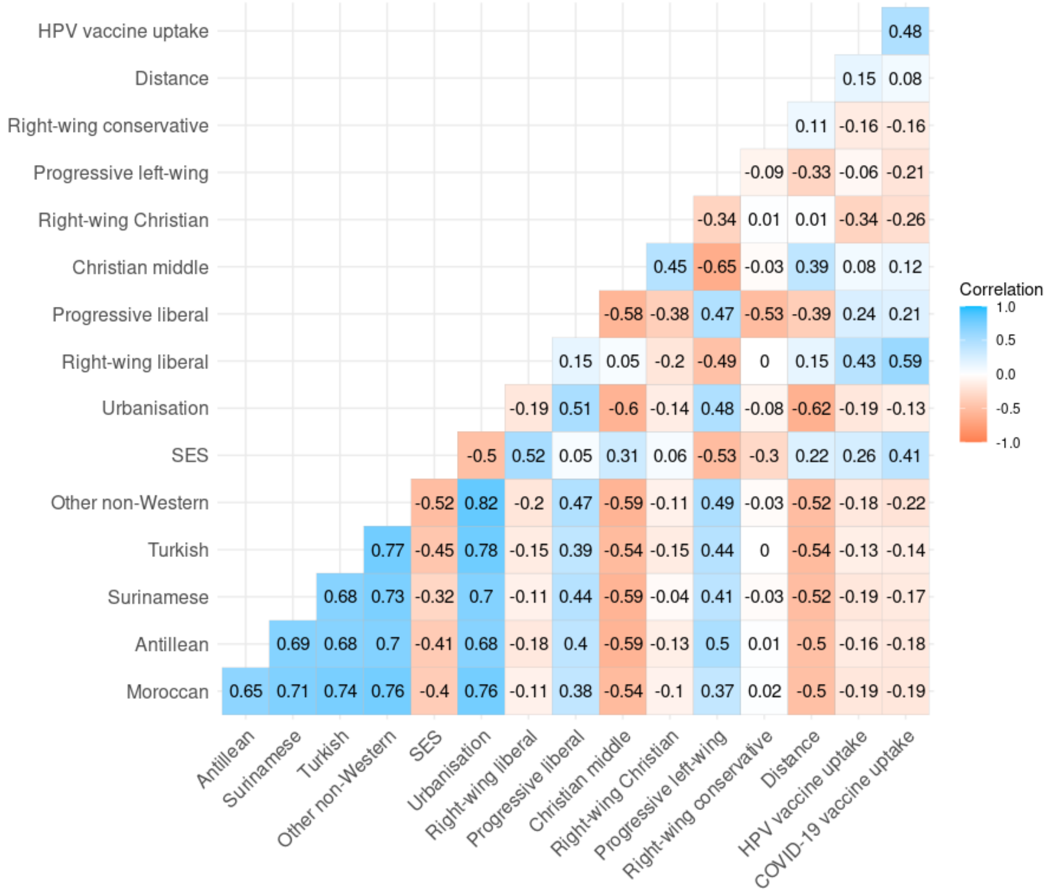

Figure S4.2. **Correlations between all potential determinants, HPV vaccine uptake and COVID-19 vaccine uptake at neighbourhood level (age group 12-49 years).**

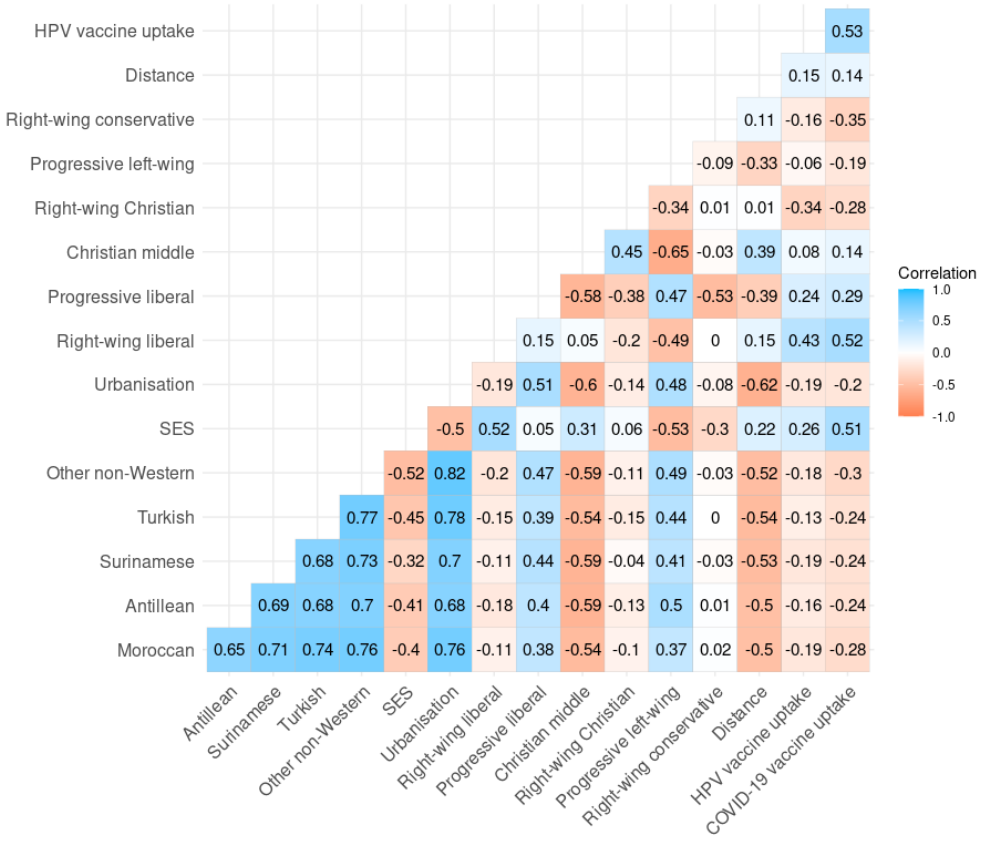

Supplement: Supplementary file 1 — Additional file 1: Table S1.1. Political parties included in the in the Dutch House of Representatives in 2021. Figure S2.1. Univariate binomial logistic regression analyses of the association at neighbourhood level between COVID-19 vaccine uptake and Moroccan, Antillean, Turkish, Surinamese, and ‘ other non-Western’ migration background, socioeconomic status score, urbanisation, distance to nearest vaccination location, voting proportions for right-wing liberal, progressive liberal, Christian middle, right-wing Christian, progressive left-wing and right-wing conservative political parties and HPV vaccine uptake. Figure S2.2. Multivariable binomial logistic regression analyses of the association at neighbourhood level between COVID-19 vaccine uptake and Moroccan, Antillean, Turkish, Surinamese, and ‘ other non-Western’ migration background, socioeconomic status score and distance to nearest vaccination location (models 1, 2 and 3). Figure S3.1. Univariate binomial logistic regression analyses of the association at neighbourhood level between COVID-19 vaccine uptake and Moroccan, Turkish, Surinamese, and ‘ other non-Western’ migration background, socioeconomic status score, urbanisation, distance to nearest vaccination location, voting proportions for right-wing liberal, progressive liberal, Christian middle, right-wing Christian, progressive left-wing and right-wing conservative political parties and HPV vaccine uptake (age group 12-49 years). Figure S3.2. Multivariable binomial logistic regression analyses of the association at neighbourhood level between COVID-19 vaccine uptake and Moroccan, Antillean, Turkish, Surinamese, and ‘ other non-Western’ migration background, socioeconomic status score, distance to nearest vaccination location and voting proportion for progressive left-wing political parties (models 1, 2 and 3) (age group 12-49 years). Figure S4.1. Correlations between all potential determinants, HPV vaccine uptake and COVID-19 vaccine uptake at neighbou [file 12889_2023_16600_MOESM1_ESM.pdf]
